# A Modified Endoscopic Transforaminal Lumbar Interbody Fusion Technique: Preliminary Clinical Results of 96 Cases

**DOI:** 10.3389/fsurg.2021.676847

**Published:** 2021-10-22

**Authors:** Junfeng Gong, Zheng Huang, Huan Liu, Chao Zhang, Wenjie Zheng, Changqing Li, Yu Tang, Yue Zhou

**Affiliations:** Department of Orthopedics, Xinqiao Hospital, Army Medical University, Third Military Medical University, Chongqing, China

**Keywords:** percutaneous endoscopic spine surgery, minimally invasive spine surgery, lumbar interbody fusion, degenerative spinal disease, PEEK cage

## Abstract

**Background:** As a newly emerging technique, endoscopic transforaminal lumbar interbody fusion (Endo-TLIF) has become an increasingly popular procedure of interest. The purpose of this study was to introduce a modified Endo-TLIF system and share our preliminary clinical experiences and outcomes in treating lumbar degenerative disease with this procedure.

**Methods:** Ninety-six patients (thirty-seven men and fifty-nine women; mean age 55.85 ± 11.03 years) with lumbar degenerative diseases who underwent Endo-TLIF in our hospital were enrolled. The surgical time, volume of intraoperative blood loss, postoperative hospitalization time and postoperative drainage were documented. Clinical outcomes were evaluated by visual analog scale (VAS) scores, Oswestry Disability Index (ODI) scores, and modified MacNab criteria. Bone fusion was identified through computerized tomography (CT) scans or X-ray during the follow-up period.

**Results:** All patients were followed up for at least 12 months, and the average follow-up time was 17.03 ± 3.27 months. The mean operative time was 136.79 ± 30.14 minutes, and the mean intraoperative blood loss was 53.06 ± 28.89 ml. The mean VAS scores of low back pain and leg pain were 5.05 ± 1.37 and 6.25 ± 1.03, respectively, before surgery, which improved to 2.27 ± 0.66 and 2.22 ± 0.55, respectively, after the operation (*P* < 0.05). The final VAS scores of low back pain and leg pain were 0.66 ± 0.60 and 0.73 ± 0.66, respectively (*P* < 0.05). The preoperative ODI score (49.06 ± 6.66) also improved significantly at the 3-month follow-up (13.00 ± 7.37; *P* < 0.05). The final ODI score was 8.03 ± 6.13 (*P* < 0.05). There were 10 cases of non-fusion (nine women and one man) at the 12-month follow-up, but no cases of non-union were identified by imaging at the final follow-up.

**Conclusions:** The present study demonstrated satisfactory clinical and radiologic results among patients who received Endo-TLIF treatment from our institution. This indicates that Endo-TLIF is efficient and safe for select patients.

## Introduction

Since Bagby first described interbody spine fusion with cages (1), spinal fusion has been an effective therapy for various lumbar degenerative disorders. Minimally invasive spine techniques, including minimally invasive transforaminal lumbar interbody fusion (MIS-TLIF), anterior lumbar interbody fusion (ALIF), and oblique lumbar interbody fusion (OLIF), have become increasingly popular over the past decade, with benefits of less blood loss, shorter hospitalization, and faster recovery over more invasive procedures. Previous reports presented good clinical outcomes in treating spine degenerative diseases (2–4). With the increasing development of endoscopic techniques and surgical instruments, endoscopic transforaminal lumbar interbody fusion (Endo-TLIF) techniques have been introduced in the field of lumbar fusion surgery and have achieved good clinical outcomes (5–8). Endo-TLIF is performed through a transforaminal approach, a well-known pathway that allows direct access to the interbody space with little bone and tissue removal. However, a steep learning curve and high rate of complications, including radicular injury, cage migration, and long duration before spine fusion, have resulted in a low level of enthusiasm for this approach by spine surgeons in the past decade. Said et al. reported the clinical and radiologic results of 60 patients treated with Endo-TLIF, and the complication rate reached 20% (9). Morgenstern et al. reported 51 cases of percutaneous transforaminal lumbar fusion, and the nerve complication rate reached 27%, including 12 patients with ipsilateral dysesthesia and 2 with ipsilateral muscle weakness (10). Therefore, it is essential to continue to develop and improve this technique.

Based on research about the anatomy of Kambin's triangle, a novel percutaneous endoscopic lumbar interbody fusion system was designed. The key part of this system is that there is a tubule that can dilate caudally during cage insertion, enlarging the working space. The purpose of this study was to introduce a modified Endo-TLIF system designed by our department and share our preliminary clinical experiences with and outcomes of the procedure in treating lumbar degenerative disease.

## Materials and Methods

### Patients

This is a retrospective study of an innovative tool applied to treat lumbar degenerative diseases with Endo-TLIF. Ninety-six patients (thirty-seven men and 59 women; mean age 55.85 ± 11.03 years) with lumbar degenerative diseases who underwent Endo-TLIF in our hospital were retrospectively enrolled. Among all the cases, a 15-year-old young man (BMI = 31.9) received fusion surgery. He was diagnosed with Meyerding grade II spondylolisthesis one year prior and received conservative treatment. However, the low back pain was not relieved, and we decided to perform lumbar fusion.

The surgical indications for Endo-TLIF included lumbar spondylolisthesis (below Meyerding grade II), lumbar instability, lumbar spinal nerve canal stenosis, lumbar discogenic pain and recurrent lumbar disc herniation. The spinal levels ranged from L3-4 to L5-S1: L3-4 for six patients, L3-5 for five patients, L4-5 for 75 patients, and L5-S1 for 10 patients (Table 1). This study was approved by the Medical Ethics Committee of the Second Affiliated Hospital of Army Medical University and was conducted according to the Declaration of Helsinki. All patients were informed of all potential outcomes of the procedure and signed written consent preoperatively.

**Table 1:** Basic demographic and surgical characteristics.

**Characteristic**	**Value**
Age(years)	
Mean	55.85 ± 11.03
Range	15–77
Sex	
Male	37
Female	59
Diagnosis	
Degenerative spondylolisthesis	47
Lumbar spinal canal stenosis	14
Segmental instability	12
Recurrent lumbar disc herniation	8
Lumbar discogenic pain	8
Isthmic spondylolisthesis	7
Surgical levels	
L3–4	6
L3–5	5
L4–5	75
L5–S1	10
Operation time(mins)	136.79 ± 30.14
Intraoperative blood loss(ml)	53.06 ± 28.89
Postoperative drainage(ml)	40.26 ± 11.75
Postoperative hospitalization time(days)	3.51 ± 0.89

All patients were definitively diagnosed *via* X-rays, magnetic resonance imaging (MRI), and computerized tomography (CT) in conjunction with clinical symptoms and signs. All patients underwent unsatisfactory conservative treatment and required surgical treatment. To evaluate the surgery, we collected and documented the surgical time, volume of intraoperative blood loss, postoperative hospitalization time and total postoperative drainage. Visual Analog Scale (VAS) scores for low back pain and leg pain before surgery, 1 day after the operation, 3 months after the operation and at the final follow-up were used to assess the clinical outcomes of the patients. Oswestry Disability Index (ODI) scores were obtained preoperatively, 3 months postoperation and at the last follow-up. The results of the procedure were classified as excellent, good, fair, and poor by using the modified MacNab criteria (11) according to patient satisfaction at the final follow-up. Postoperative follow-up images, such as X-ray and CT images, were assessed by an orthopedic surgeon, and the status of interbody fusion was graded by the Brantigan, Steffee, Fraser—BSF—classification system: BSF-1 (radiographic pseudarthrosis), BSF-2 (radiographic locked pseudarthrosis) and BSF-3 (radiographic fusion) (12, 13).

### Surgical Technique

Endo-TLIF surgery is a development of the percutaneous endoscopic lumbar discectomy (PELD) technique, in which lumbar interbody fusion is manipulated through a transforaminal approach. Kambin's triangle is the working zone of Endo-TLIF, defined by 3 borders: the bottom is the superior endplate of the caudal vertebra, the height is the traversing nerve root, and the hypotenuse is the exiting nerve root. All the procedures are achieved in the theoretical triangle.

**Figure 1:**
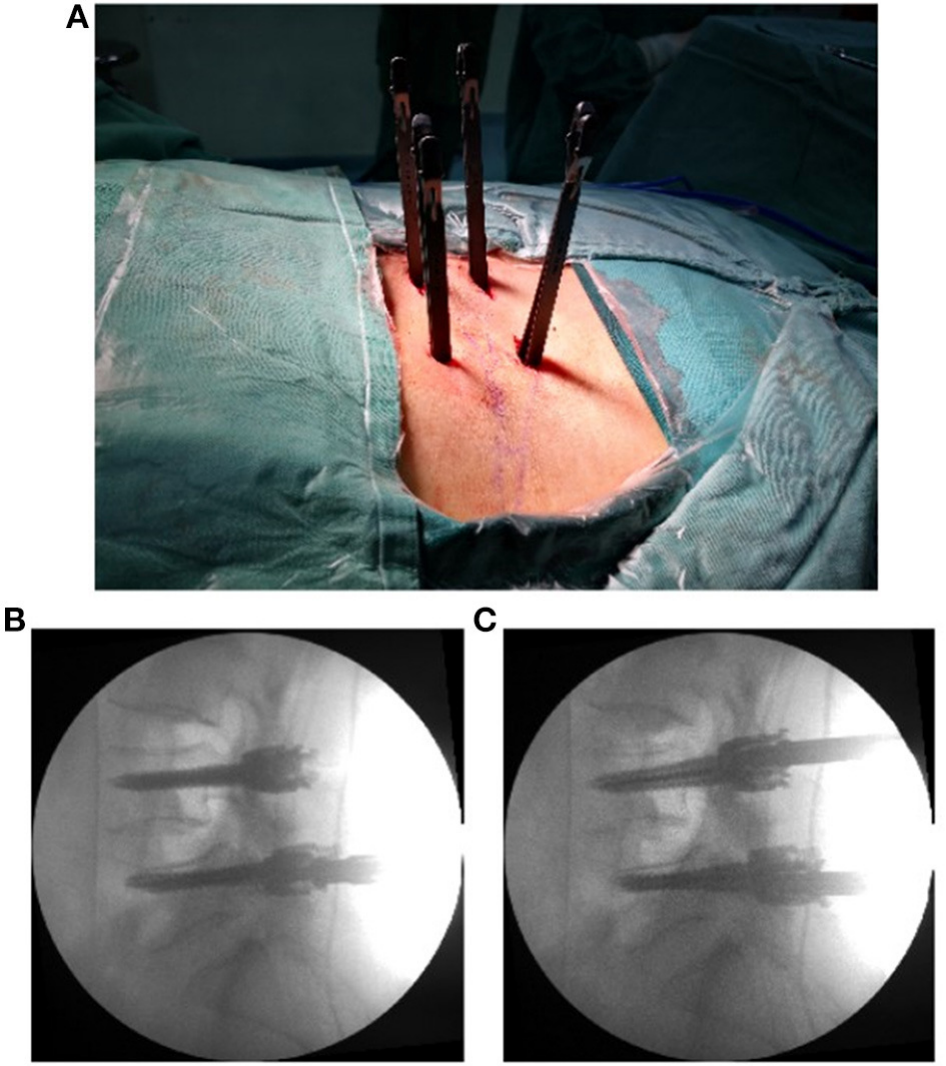
The pedicle screws were placed percutaneously **(A)**. The positions of the screws are shown in the X-ray lateral view **(B)**, and the intervertebral foramen was enlarged by a pedicle screw distractor **(C)**.

**Figure 2:**
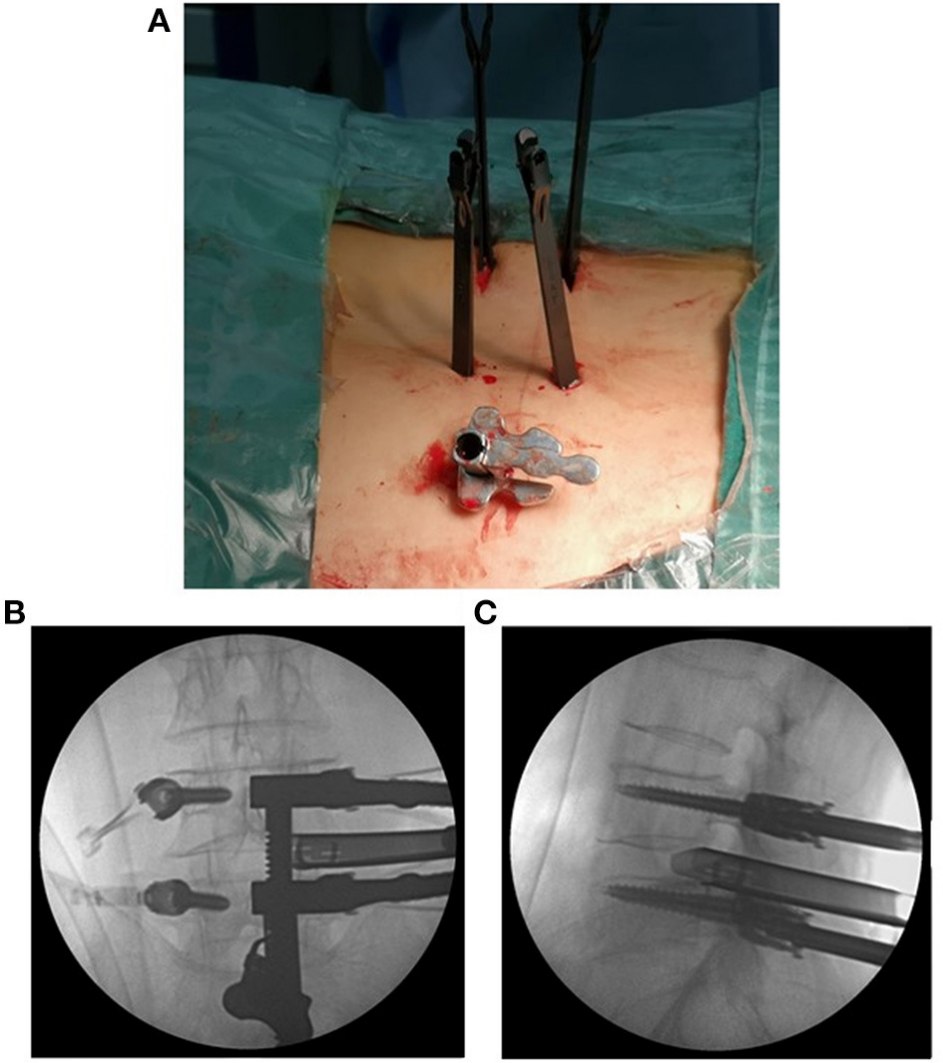
The expandable tube was positioned at a suitable location **(A)**. The anteroposterior and lateral views of the positioning of the expandable tube are shown **(B,C)**.

**Figure 3:**
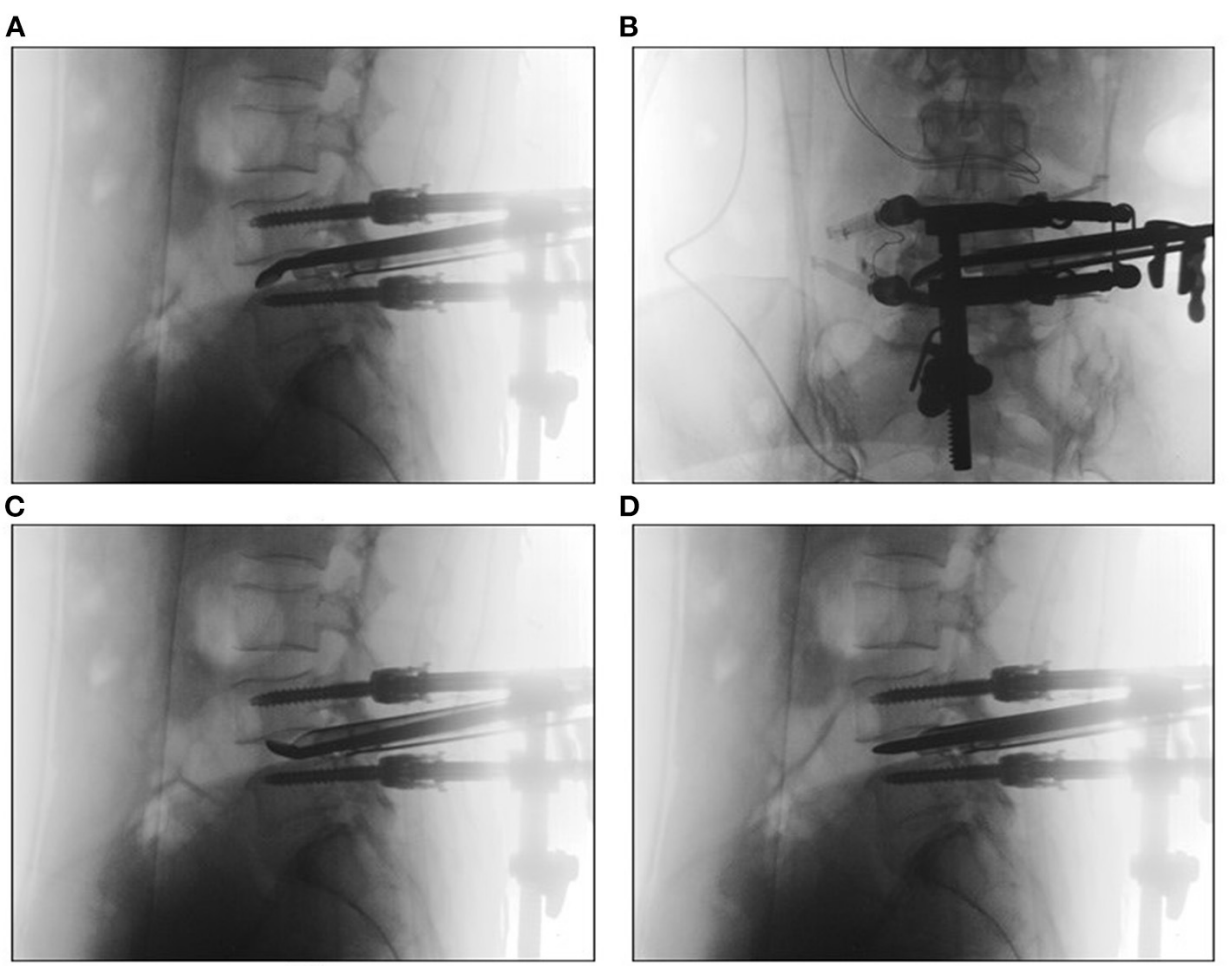
Endplate preparation was performed with various tools through an expandable tube **(A–D)**.

**Figure 4:**
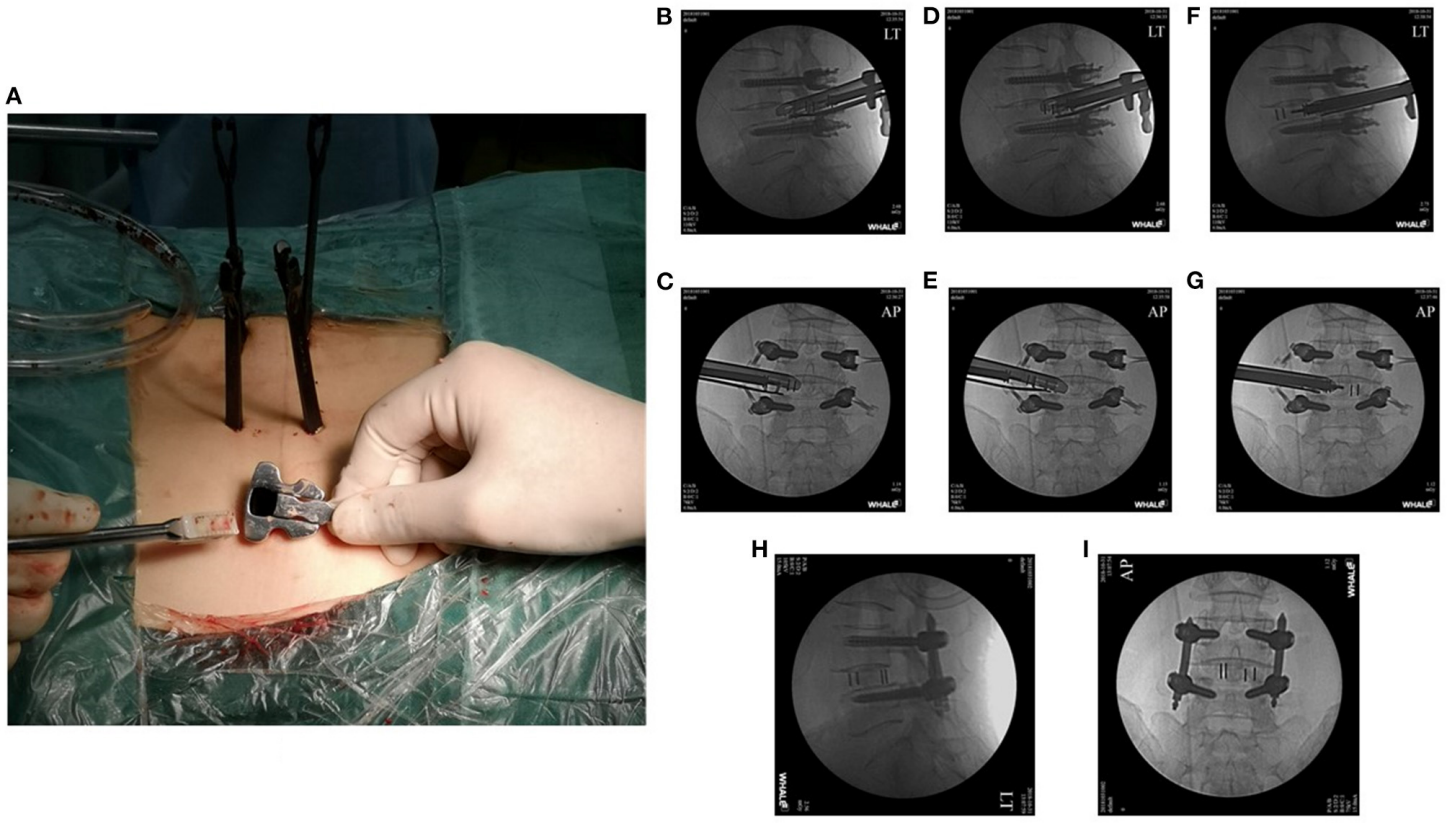
A conventional cage was placed through the working tube under fluoroscopic guidance **(A–G)**. The final cage position is shown in the anteroposterior and lateral X-rays **(H,I)**.

The operation was performed under general anesthesia, with the patient positioned prone on a radiolucent table. A neurological monitoring system was used to monitor somatosensory evoked potentials and free-running electromyography during the whole procedure. After the operated segments were confirmed by G-arm fluoroscopy, pedicle screws were placed percutaneously at the diseased segments. Then, a pedicle screw distractor was used to enlarge Kambin's triangle to perform the subsequent procedures more conveniently (Figure 1). The working channel position on the entry point on the skin was ~6–8 cm from the midline at a 45°-55° angle to the horizon. A spinal needle was used to access the intervertebral foramen and was then replaced by a 0.8 mm guidewire. If the needling target point was unsatisfactory, targeted foraminoplasty (14) was performed. Next, variously sized dilators were progressively introduced along the guidewire, and finally, a C-shaped tubule with an opening at the caudal side was positioned at a suitable location under fluoroscopic guidance (Figure 2). A flexible baffle was pushed down along the distal side of the tubule. When construction of the expandable tube was completed, an intervertebral space dilator was inserted into the disc space and rotated back and forth to create sufficient space for the implant. Curettes, reamers, pituitary rongeurs, and raspatories were used to prepare the space for the endplate through the expandable tube (Figure 3). Thereafter, allografts (4–8 g) and recombinant human bone morphogenetic protein (rhBMP) were placed into the anterior intervertebral disc through a funnel-shaped bone graft device. A standard rigid cage filled with allograft was inserted under neuromonitoring through the expandable working tube. The final cage position was confirmed by X-ray (Figure 4).

After cage placement, the expandable tube was replaced by an endoscopic working channel. Through this working channel, sufficient neural decompression was performed *via* a standard PELD procedure. Cage position and neural decompression were confirmed again by endoscopy. Then, pedicle screws were fixed bilaterally with connecting rods. Following final tightening and locking of the set screws, the small incisions were directly closed, and subfascial wound drainage was installed.

### Statistical Analysis

Measurement data are expressed as the mean ± standard deviation (SD). Statistical analysis of the data was conducted using SPSS 23.0 software, and significant differences in repeated-measures data (VAS, ODI) were determined using repeated-measures analysis of variance. Statistical significance was set at a *P*-value <0.05.

## Results

**Figure 5:**
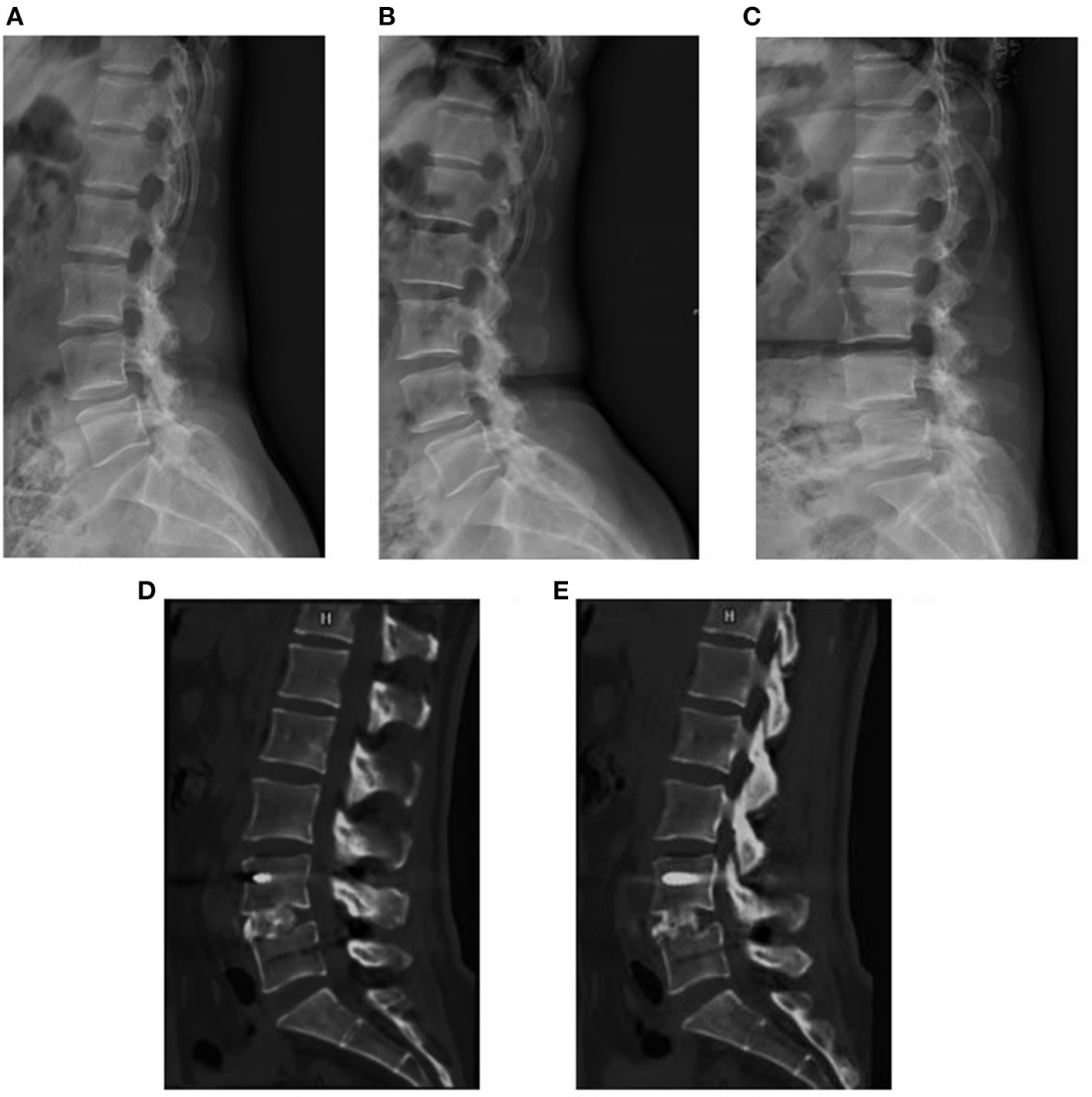
A 48-year-old woman suffered from low back pain for 6 months. The preoperative X-rays showed Meyerding grade I lumbar spondylolistheses at L4 **(A–C)**. The sagittal CT images at 7 months postoperatively suggested solid spine fusion **(D,E)**.

All patients were followed up for at least 12 months, and the average follow-up time was 17.03 ± 3.27 months. The mean operative time was 136.79 ± 30.14 minutes, and the mean intraoperative blood loss was 53.06 ± 28.89 ml. The average postoperative drainage volume was 40.26 ± 11.75 ml. The mean postoperative hospital stay was 4.69 ± 1.01 days (Table 1). The mean VAS scores of low back pain and leg pain were 5.05 ± 1.37 and 6.25 ± 1.03, respectively, which improved to 2.27 ± 0.66 and 2.22 ± 0.55 after the operation (*P* < 0.05). The VAS low back pain and leg pain scores were 1.01 ± 0.48 and 0.95 ± 0.51, respectively, 3 months after the operation, which improved significantly over the corresponding preoperative values (*P* < 0.05). The final VAS scores of low back pain and leg pain were 0.66 ± 0.60 and 0.73 ± 0.66, respectively (*P* < 0.05). The preoperative ODI score (49.06 ± 6.66) also improved significantly at the 3-month follow-up (13.00 ± 7.37; *P* < 0.05). The final ODI score was 8.03 ± 6.13 (*P* < 0.05). According to the modified MacNab criteria at the final follow-up, 84 patients were regarded as having excellent clinical outcomes, 10 patients as having good clinical outcomes, one patient as having a fair clinical outcome, and one patient was regarded as having a poor outcome (Table 2). There were 10 cases of non-fusion (nine women and one man) at the 12-month follow-up, and the reason may be related to osteoporosis, diabetes mellitus and high-risk factors for non-fusion, resulting in delayed fusion. However, all cases showed good spine fusion with BSF-3 at the last follow-up period, and there were no clinical symptoms of non-union, such as worsening axial pain. A representative case is shown in Figure 5.

**Table 2:** Follow-up outcomes (VAS, ODI, modified Macnab criteria).

**Characteristic**	**Value**
**VAS scores of low back pain**	
Preoperative	5.05 ± 1.37
Postoperative	2.27 ± 0.66^*^
3 months	1.01 ± 0.48^*^
Final follow-up	0.66 ± 0.60^*^
**VAS scores of leg pain**	
Preoperative	6.25 ± 1.03
Postoperative	2.22 ± 0.55^*^
3 months	0.95 ± 0.51^*^
Final follow-up	0.73 ± 0.66^*^
**ODI scores**	
preoperative	49.06 ± 6.66
3 months	13.00 ± 7.37^*^
Final follow-up	8.03 ± 6.13^*^
**Modified Macnab criteria,n**	
Excellent	84
Good	10
Fair	1
Poor	1

* *Significantly different from the preoperative value (P < 0.05)*.

Two cases of dural tears and two cases of temporary ipsilateral dysesthesia were observed. Most clinical signs of ipsilateral dysesthesia disappeared within 1–3 days after the operation. However, of the two patients with temporary ipsilateral dysesthesia, one suffered radiculitis, and conservative treatment was ineffective. Finally, he recovered after receiving an additional nerve root block with 2% lidocaine and betamethasone. Only one patient experienced cage migration and therefore received a revision operation. No epidural hematomas, infection, or muscle paralysis were observed among our patients.

## Discussion

Various minimally invasive fusion surgeries, such as MIS-TLIF, ALIF, and OLIF, have been performed to minimize the procedure-related destruction of spinal muscles and ligaments (2–4). Such procedures can be practical alternatives to conventional open surgeries. As a newly emerging technique, Endo-TLIF has become an increasingly popular procedure of interest, having advantages such as a quick recovery, reduction in postoperative back pain, and little soft tissue destruction. When Endo-TLIF is performed through Kambin's triangle, normal structures such as the facets, muscle, and ligaments can be preserved maximally. In general, taking the size of the implant cage into consideration is important when performing the operation. The box-shaped polyetheretherketone (PEEK) cage currently in use is too large to be placed *via* an endoscopic working tube. A large-diameter tube may be helpful but places the dorsal root ganglion at increased risk of injury. In 2013, Jacquet et al. reported 57 patients who underwent Endo-TLIF; the outcome was not satisfactory because of high complication rates, and the authors suggested making decisive technical improvements (6). With further improvements in the associated surgery and tools, Endo-TLIF has achieved varying degrees of success in subsequent reports (5, 7, 8, 15).

**Figure 6:**
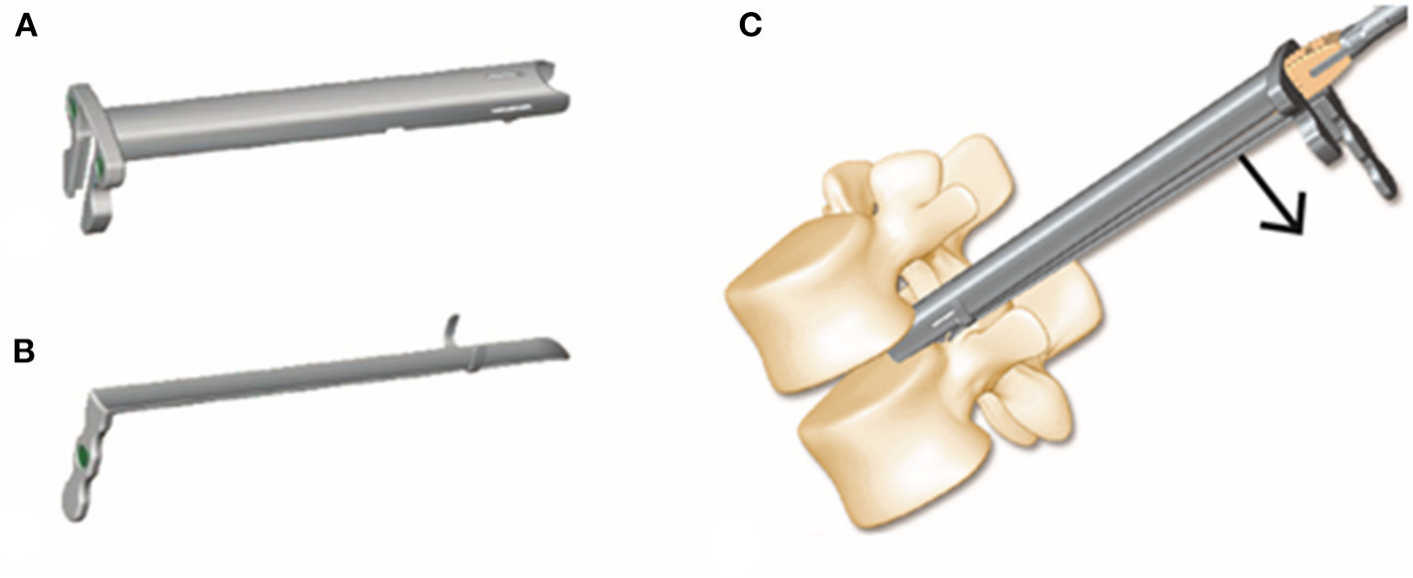
The expandable tube consists of a rigid tubule with an opening at the distal side and a flexible baffle **(A,B)**. The tube expands when the cage is inserted, establishing a safe working space **(C)**.

Establishing a safe working pathway for percutaneous access to the disc is crucial to Endo-TLIF. Kambin's triangle (16, 17) is a safer corridor for access into the disc than the posterolateral disc. Although the safe working zones for Endo-TLIF are the same as those utilized for PELD, the goals of the two techniques are substantially different. To achieve spine fusion in Endo-TLIF, the endplate is prepared with various instruments, and the implant is placed through a working tube. These procedures could increase the risk of injury to the exiting nerves; therefore, we designed a new Endo-TLIF system (ZELIF®, Sanyou, Inc., Shanghai, China). The key part of this novel system is an expandable tube composed of two parts, a rigid C-shaped tubule with an opening at the distal side covered by a flexible baffle. The expandable tube can dilate during the cage insertion procedure, establishing a larger working space through which the cage can be inserted safely (Figure 6). In a previous report, the placement of conventional PEEK implants was difficult during Endo-TLIF because of the limited size of the working channel. As a result, expandable cages have been used in most subsequent Endo-TLIF surgeries (5, 18, 19). However, the supportive load of expandable cages remains to be further studied. By utilizing this new Endo-TLIF system, conventional open reamers and curettes can be used for endplate preparation, and a rigid PEEK cage (from 12 to 14 mm) can be inserted into the disc. Compared with expandable cages, the placement of conventional PEEK cages can reduce complications such as cage migration and subsidence, increasing the success rate of spine fusion; additionally, it can reduce the load on the pedicle screw fixation system and redistribute the endplate stress, providing sufficient spine stability (20). Meanwhile, indirect neural decompression can be achieved through disc height restoration and intervertebral foramen expansion by inserting a large, conventional PEEK cage (21). However, the small amount of autogenous bone removed during the procedure is insufficient for filling large cages. rhBMP and allografts (4–8 g) are typically used in our institution instead.

In the present study, combined with percutaneous posterior fixation and placement of conventional rigid PEEK implants, Endo-TLIF treatment resulted in satisfactory surgical outcomes among the patients in our institution. Many patients reported that their low back pain and/or leg pain were substantially relieved, with no need for the use of opioids postoperatively. All patients were able to sit up in bed immediately after surgery and stand up 2–3 days post-operation. Perioperative complications appeared in five patients (dural tears in two patients, cage migration in one patient, and temporary ipsilateral dysesthesia in two patients), which occurred mainly early in the implementation of Endo-TLIF. Dural tears occurred in those who received revision spine surgery because the adhesion between scar tissue and the dura was severe. In general, dural tears or defects are small, and they can be resolved by conservative management, such as bed rest. Placement of a small cage (11 mm in size) and strenuous activity in the early postoperative period resulted in cage migration. To prevent the occurrence of cage migration, it is necessary to place as large a cage as possible. Outward movement of the expandable tube, which caused irritation of the exiting nerve, was responsible for temporary ipsilateral dysesthesia. Hence, the tubule should be docked firmly into the disc space under fluoroscopy to avoid irritation of the nerve.

Endo-TLIF also has some limitations. First, as with other endoscopic surgeries, it has a steep learning curve that requires proper training; we recommend performing Endo-TLIF after becoming skillful in PELD. Next, intraoperative medical procedure exposure to X-rays remains a problem. Intraoperative CT combined with a navigation system may be useful in improving the efficiency of the surgery and reducing the exposure of medical workers (22, 23). In our experience, some key points should be considered in performing Endo-TLIF. (1) Low exiting nerve roots or exiting nerve root variability should be carefully identified through CT or MRI, and magnetic resonance neurography is an adequate technology for the analysis of the safety of Endo-TLIF (24). (2) sufficient disk removal and endplate preparation are important for spine fusion. (3) this technique is not recommended if the patient has Meyerding grade II or above lumbar spondylolistheses or severely deformed intervertebral foramina.

To our knowledge, there are few reports about the use of large conventional cages in Endo-TLIF. The major advantage of our study is that we designed a new Endo-TLIF system that maximally preserves the normal spine structure, improves the safety of the procedure and implants a large conventional PEEK cage.

## Conclusions

The present study illustrated satisfactory clinical and radiologic results among patients who received Endo-TLIF treatment in our institution. This indicates that Endo-TLIF is efficient and safe for select patients. A long-term follow-up study involving a larger cohort should be performed to further assess the clinical results of this new technique in the future.

## Data Availability Statement

The raw data supporting the conclusions of this article will be made available by the authors, without undue reservation.

## Ethics Statement

The studies involving human participants were reviewed and approved by the Medical Ethics Committee of the Second Affiliated Hospital of Army Medical University. The patients/participants provided their written informed consent to participate in this study. Written informed consent was obtained from the individual(s) for the publication of any potentially identifiable images or data included in this article.

## Author Contributions

JG and ZH: conceptualization and data curation. JG: methodology and formal analysis. HL: software. ZH, YT, and YZ: validation. ZH: investigation. CZ, WZ, and CL: resources. JG, ZH, and HL: writing—original draft preparation. YT and YZ: writing—review and editing. JG, CL, and YT: visualization. CL and YZ: supervision. CL: project administration. All authors have read and agreed to the published version of the manuscript.

## Funding

This work was supported by the Chongqing technological innovation and application development project (Grant No. cstc2019jscx-msxmX0160).

## Conflict of Interest

The authors declare that the research was conducted in the absence of any commercial or financial relationships that could be construed as a potential conflict of interest.

## Publisher's Note

All claims expressed in this article are solely those of the authors and do not necessarily represent those of their affiliated organizations, or those of the publisher, the editors and the reviewers. Any product that may be evaluated in this article, or claim that may be made by its manufacturer, is not guaranteed or endorsed by the publisher.
